# Feeding intolerance and risk of poor outcome in patients undergoing cardiopulmonary bypass surgery

**DOI:** 10.1017/S0007114521000167

**Published:** 2021-11-14

**Authors:** Yanjuan Lin, Meihua Chen, Yanchun Peng, Qiong Chen, Sailan Li, Liangwan Chen

**Affiliations:** 1 Department of Nursing, Fujian Medical University Union Hospital, 29 Xinquan Road, Fuzhou City, Fujian Province, People’s Republic of China; 2 Fujian Medical University, 88 Jiaotong Road, Fuzhou City, Fujian Province, People’s Republic of China; 3 Department of Cardiac Surgery, Fujian Medical University Union Hospital, 29 Xinquan Road, Fuzhou City, Fujian Province, People’s Republic of China

**Keywords:** Cardiopulmonary bypass surgery, Enteral nutrition, Feeding intolerance, Poor prognosis

## Abstract

We conducted a prospective, observational study to determine the incidence of feeding intolerance (FI) within 7 d of initiating enteral nutrition (EN) in patients undergoing cardiopulmonary bypass (CPB) and to evaluate the association between FI and a poor prognosis. Patients who underwent CPB surgery at Fujian Medical University Union Hospital between March 2020 and June 2020 were enrolled. According to the presence or absence of FI within 7 d after EN, patients were divided into FI and non-FI groups. According to the occurrence of a poor prognosis (death, gastrointestinal haemorrhage, acute kidney injury, liver insufficiency, neurological events (cerebral infarction, cerebral haemorrhage and epilepsy) and prolonged mechanical ventilation (> 48 h)), patients were divided into poor prognosis and good prognosis groups. The mean age of the 237 CPB patients, including 139 men and ninety-eight women, was 53·80 (sd 12·25) years. The incidence of FI was 64·14 %. Multivariate logistic regression analysis showed factors independently associated with poor prognosis after CPB included FI (OR 2·138; 95 % CI 1·058, 4·320), age (OR 1·033; 95 % CI 1·004, 1·063), New York Heart Association (NYHA) class III/IV cardiac function (OR 2·410; 95 % CI 1·079, 5·383), macrovascular surgery (OR 5·434; 95 % CI 1·704, 17·333) and initial sequential organ failure assessment score (OR 1·243; 95 % CI 1·010, 1·530). Thus, the incidence of FI within 7 d of EN after CPB was high, which was associated with a poor prognosis.

The 2019 guidelines on enteral nutrition (EN) by the European Society for Clinical Nutrition and Metabolism (ESPEN) state that patients who can fully adapt to EN and have strong recovery ability should receive EN^(1)^. However, feeding intolerance (FI) during EN, especially after cardiopulmonary bypass (CPB), can impact nutritional support programmes significantly^(2)^.

During CPB, redistribution of blood flow to critical body organs, such as the brain, results in earlier reduction in blood flow to abdominal organs. Moreover, other factors, such as haemodilution, cause the gastrointestinal tract to be in a state of low perfusion and hypoxia, which leads to functional gastrointestinal injury^(3,4)^.

FI is the most common clinical manifestation of functional gastrointestinal injury; a higher incidence of gastrointestinal functional injury is associated with a higher incidence of FI^(5,6)^. According to the literature, the incidence of FI in critically ill patients during EN is between 30·5 and 65·7 %, which causes critically ill patients to fail to meet their nutritional goals, prolongs the duration of mechanical ventilation and increases the length of stay at the intensive care unit (ICU)^(2,7)^. A case–control retrospective study reported that FI within 3 d of admission to the neurological ICU was an independent predictor of poor prognosis in patients with severe neurological diseases^(8)^. The contribution of malnutrition to a poor prognosis has increasingly attracted the attention of medical practitioners. The present research has, however, focused mainly on FI in critically ill patients after non-cardiac surgery and pre-mature infants, with relatively fewer reports on the incidence of FI and whether FI affects the prognosis in CPB patients. Therefore, we aimed to determine the incidence of FI and to investigate whether FI is associated with a poor prognosis in CPB patients.

## Experimental methods

### Participants and study design

In this prospective, observational study, CPB patients aged >18 years admitted to the Fujian Medical University Union Hospital from March 2020 to June 2020 were included. According to the definition of FI, whereby one or more of the following features are present: large gastric residual volume, abdominal distension, vomiting, diarrhoea or constipation^(2)^, patients were divided into FI and non-FI groups. Patients with an ICU duration of stay of < 24 h, gastrointestinal tumours, chronic diarrhoea, total intestinal obstructions, gastrointestinal resections on admission, and in whom the initial EN time was longer than 48 h after operation and patients with serious mental illnesses were excluded from the study.

All patients received appropriate fluid resuscitation and ventilation therapy. Routine conditions for EN include a semi-decubitus position between 30° and 45°. Patients were assigned to receive either a nasogastric tube EN or an oral EN. Oral intake was performed based on the patient having removed the endotracheal tube and no swallowing disturbance, the gastric tube was indwelling at the bedside, and the position of the gastric tube was determined by the sound of air and water and PH. Gastrointestinal motility drugs were added when the gastric residual amount was more than 200 ml or when abdominal distension occurred. When the patient was constipated, laxative treatment was administered; when the patient had diarrhoea, antidiarrhoeal treatment was administered. According to our ICU mechanical ventilation guidelines, all patients receive the same care. When the extubation standard was reached, mechanical ventilation of the patient ceased.

### Ethical approval

The present study was conducted according to the guidelines laid down in the Declaration of Helsinki, and all procedures involving human subjects/patients were approved by the ethics committee of the Fujian Medical University Union Hospital (ethics number: 2020KY014). Written informed consent was obtained from all participants.

### Variables and data collection

A data collection table was designed by the researchers. Partial data were prospectively collected by a stationary researcher using the hospital clinical electronic database. Data collected included demographic characteristics (age, sex and BMI), education level, marital status, lifestyle (smoking and alcohol history) and past medical history (hypertension, diabetes and cerebral infarction)). The clinical data included preoperative data (New York Heart Association (NYHA) cardiac functional classification), intraoperative data (type of cardiac surgery, operation time, CPB time and aortic clamping time), postoperative data (sequential organ failure assessment (SOFA) score on the first day after surgery, the highest vasoactive-inotropic score (VIS) within 24 h after surgery, the highest lactic acid score within 24 h after surgery, the MAP within 24 h after surgery, duration of mechanical ventilation, length of ICU and hospital stay), relevant data on FI (vomiting, gastric residual volume, diarrhoea, abdominal distension and constipation) and EN-related data (type, quantity and mode).

### Outcome indicators

The main outcome indicators were death during hospitalisation, postoperative complications, including gastrointestinal haemorrhage, acute kidney injury, liver insufficiency, neurological events (cerebral infarction, cerebral haemorrhage and epilepsy) and long-term mechanical ventilation.

### Definition of variables

FI within 7 d after EN: the patient had one or more of the following symptoms within 7 d after receiving EN, including vomiting, diarrhoea, abdominal distension, constipation or large gastric residues^(2)^. Vomiting was defined as the reflux of any visible gastrointestinal content regardless of the amount of vomit (except vomiting due to mechanical movement of the endotracheal tube and nasogastric tube). Diarrhoea was defined as an increase in faecal moisture and stool frequency; frequency of defecation > 3 times/d or faecal volume > 200 g/d. A large gastric residual volume was defined as a single gastric residue withdrawal of > 200 ml. Abdominal distension was defined as abdominal distension or fullness discomfort, which could be subjective. Liver insufficiency was defined as a peak serum total bilirubin level of > 7 mg/dl after surgery^(9)^. Prolonged mechanical ventilation was defined as ventilation for ≥ 48 h^(10)^. According to The Kidney Disease: Improving Global Outcomes (KDIGO) criteria, AKI was defined as any of the following: an increase in serum creatinine by ≥ 0·3 mg/dl (≥ 26·5 µmol/l) within 48 h; or increase in serum creatinine to ≥ 1·5 times baseline level within 7 d after surgery; or urine volume < 0·5 ml/kg per h for 6 h^(11)^. Poor prognosis was defined as the occurrence of gastrointestinal haemorrhage, acute kidney injury, liver insufficiency, neurological events (cerebral infarction, cerebral haemorrhage and epilepsy) or prolonged mechanical ventilation.

The SOFA score used the worst value of each parameter within 24 h of the operation. The VIS score and lactic acid value were the highest values within 24 h after surgery. For the calculation of the vascular active drug usage peak 24 h after surgery reports, the Gaies^(12)^ formula for the determination of the VIS value was used: VIS = dopamine (μg/kg per min) + dobutamine (μg/kg per min) + 10 × milrinone (μg/kg per min) + 100 × adrenaline (μg/kg per min) + 100 × norepinephrine (μg/kg per min) + 10 000 × pituitrin (U/kg per min). All vasoactive drugs were continuously pumped through a micropump.

### Statistical analyses

All data were analysed using SPSS 25.0 statistical software (SPSS Inc.). Normally distributed data are described as means and standard deviations, and the group *t* test was used for comparison between groups. Non-normally distributed data are represented by medians and interquartile ranges (P25, P75), and the rank sum test was used for comparison between the groups. The count data are described by frequencies and percentages; the *χ* ^2^ test or Fisher’s exact test was used for comparison between groups. Univariate logistic regression analysis was used to identify risk factors associated with poor prognosis, and multivariate logistic regression analysis was conducted to determine significant risk factors. *P* values < 0·05 were considered statistically significant.

## Results

### Selection of participants

**Fig. 1.**
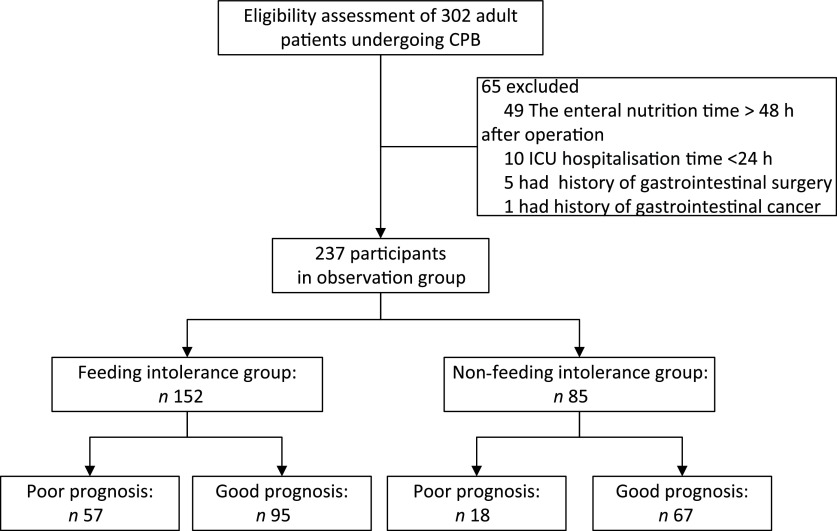
Inclusion of study participants. CPB, cardiopulmonary bypass; ICU, intensive care unit.

The inclusion of participants in the study is summarised in Fig. 1. Of the 302 patients enrolled from 1 March 2020 to 30 June 2020, after applying the exclusion criteria, 237 were included in the analysis. There were 152 patients in the FI group and eighty-five in the non-FI group. The incidence of FI was 64·14 %.

### Demographic and clinical characteristics of patients with and without feeding intolerance

Tables 1 and 2 show the demographic and clinical characteristics of patients in the two groups. The mean age of the 237 CPB patients, including 139 men (58·6 %) and ninety-eight women (41·4 %), was 53·80 (sd 12·25) years. The mean SOFA score of the study participants was 11·82 (sd 1·914). Compared with the non-FI group, the FI group had a significantly longer operation time (251 *v.* 221 min, *P* = 0·014), CPB time (122 *v.* 101 min, *P* = 0·001), aortic clamping time (62·5 *v.* 52 min, *P* = 0·023) and length of hospital stay (20 *v.* 18 d, *P* = 0·020).

**Table 1.** Comparison of baseline data between the feeding intolerance (FI) and non-FI groups (Mean values and standard deviations; numbers and percentages)

Variable	Total (*n* 237)		Non-FI group (*n* 85)		FI group (*n* 152)		*P*
	*n*	%	*n*	%	*n*	%	
Demographic factors							
Age (years)							0·441
Mean	53·80		52·98		54·26		
sd	12·25		12·86		11·91		
Sex, male	139	58·65	55	64·7	84	55·2	0·157
BMI (kg/m^2^)							0·348
Mean	22·86		22·62		22·99		
sd	3·14		2·66		3·38		
Education level							0·721*
Primary schools and below	135	56·96	50	58·8	85	55·9	
Middle school	50	21·1	16	18·8	34	22·4	
High school or technical secondary school	32	13·5	14	16·5	18	11·8	
Junior college or above	20	8·44	5	5·9	15	9·8	
Smoker	63	26·6	21	24·7	42	27·6	0·625
Drinker	8	3·4	4	4·7	4	2·6	0·462*
Marital status	232	97·9	82	96·5	150	98·7	0·353*
Past medical history							
Hypertension	60	25·3	23	27·1	37	24·3	0·645
Diabetes	14	5·9	6	7·1	8	5·3	0·583
Cerebral infarction	13	5·5	2	2·4	11	7·3	0·143*

* Fisher’s exact test.

**Table 2.** Comparison of clinical data between the feeding intolerance (FI) and non-FI groups (Numbers and percentages; medians and interquartile ranges (IQR))

Variable	Total (*n* 237)		Non-FI group (*n* 85)		FI group (*n* 152)		*P*
	*n*	%	*n*	%	*n*	%	
Preoperative factors							
NYHA functional class III/IV	166	70·0	59	69·4	107	70·4	0·874
Intraoperative factors							
Macrovascular surgery	22	9·3	10	11·8	12	7·9	0·325
Operating time (min)							0·014
Median	240·00		221·0		251·0		
IQR	202·50–304·50		182·5–286·0		211·3–314·3		
CPB time (min)							0·001
Median	116·0		101·0		122·0		
IQR	83·50–159·50		68·0–141·05		93·0–163·8		
Aortic clamping time (min)							0·023
Median	60·0		52·0		62·5		
IQR	35·0–92·50		30·0–84·0		40·3–99·3		
Postoperative factors							
Length of hospital stay (d)							0·020
Median	19·0		18·0		20·0		
IQR	15·0–24·0		14·0–21·5		15·3–25·8		
Length of ICU stay (h)							0·171
Median	46		45		46		
IQR	39·0–70·0		27–69·5		40–81·8		
Mechanical ventilation time (h)							0·206
Median	20		20		20		
IQR	16·0–38·5		14·5–26·5		16–40·8		
SOFA score							0·168
Mean	11·82		11·59		11·96		
sd	1·91		1·87		2·03		
Feeding patterns							0·982
Oral feeding	195	82·3	70	82·4	125	82·2	
Nasogastric feeding	42	17·7	15	17·6	27	17·8	
VIS_max_							0·057
Median	10		10		10·5		
IQR	9–13		8–12		10–14·4		
Lactic acid_max_							0·121
Median	4·45		4·1		4·6		
IQR	2·9–6·6		2·7–6·2		2·95–6·895		
MAP							0·375
Median	84		82		84·67		
IQR	75·33–93·5		74·83–96·33		77·08–93·33		

NYHA, New York Heart Association; CPB, cardiopulmonary bypass; ICU, intensive care unit; SOFA, sequential organ failure assessment; VIS, vasoactive-inotropic score; MAP, mean arterial pressure.

### Comparison of poor prognosis between the feeding intolerance and non-feeding intolerance groups

As shown in Table 3, compared with the non-FI group, the FI group had a significantly higher proportion of patients on prolonged mechanical ventilation (>48 h) (OR 2·627; 95 % CI 1·098, 6·288), acute kidney injury rate (OR 2·569; 95 % CI 1·242, 5·310) and neurological events rate (OR 7·856; 95 % CI 1·009, 61·142). The comprehensive incidence of postoperative complications in the FI group was also higher (OR 2·233; 95 % CI 1·207, 4·132).

**Table 3.** Comparison of poor prognosis between the feeding intolerance (FI) and non-FI groups (Numbers and percentages; odd ratios and 95 % confidence intervals)

Variable	Non-FI group (*n* 85)		FI group (*n* 152)		OR	95 % CI	*P*
	*n*	%	*n*	%			
Prolonged mechanical ventilation (> 48 h)	7	8·2	29	19·1	2·627	1·098, 6·288	0·030
Acute kidney injury	11	12·9	42	27·6	2·569	1·242, 5·310	0·011
Liver insufficiency	3	3·5	5	3·3	0·930	0·217, 3·990	0·922
Death	1	1·2	6	3·9	3·452	0·409, 29·164	0·255
Neurological events*	1	1·2	13	8·6	7·856	1·009, 61·142	0·049
Gastrointestinal haemorrhage	1	1·2	6	3·9	3·452	0·409, 29·164	0·255
Overall poor prognosis	18	21·2	57	37·5	2·233	1·207, 4·132	0·010

* Cerebral infarction, cerebral haemorrhage, epilepsy.

### Univariate and multivariate comparisons of the poor prognosis group

The baseline clinical characteristics of patients with a poor prognosis are shown in Table 4. The age (OR 1·040; 95 % CI 1·015, 1·066), cerebral infarction (OR 3·725; 95 % CI 1·176, 11·806), NYHA functional class III/IV (OR 2·660; 95 % CI 1·349, 5·243), macrovascular surgery (OR 3·565; 95 % CI 1·450, 8·764), operation time (OR 1·007; 95 % CI 1·003, 1·011), CPB time (OR 1·008; 95 % CI 1·003, 1·013), SOFA score (OR 1·445; 95 % CI 1·234, 1·693), FI (OR 2·233; 95 % CI 1·207, 4·132), the highest VIS score within 24 h after surgery (OR 1·034; 95 % CI 1·008, 1·062) and the highest lactic acid score within 24 h after surgery (OR 1·109; 95 % CI 1·026, 1·198) were associated with a poor prognosis. The multivariate logistic regression analysis of poor prognosis showed that after adjusting for cerebral infarction, operating time and CPB time, the highest VIS and highest lactic acid scores within 24 h after surgery, FI (OR 2·138; 95 % CI 1·058, 4·320; *P* = 0·034), age (OR 1·033; 95 % CI 1·004, 1·063; *P* = 0·026), NYHA functional class III/IV (OR 2·410; 95 % CI 1·079, 5·383; *P* = 0·032), macrovascular surgery (OR 5·434; 95 % CI 1·704, 17·333; *P* = 0·004), and SOFA score (OR 1·243; 95 % CI 1·010, 1·530; *P* = 0·040) retained statistical significance, indicating that these variables were predictors of poor prognosis after CPB.

**Table 4.** Univariate and multivariate comparison of the poor prognosis group after cardiopulmonary bypass (CPB) (Odds ratios and 95 % confidence intervals)

Variable	Univariate analysis		Multivariate analysis	
	OR	95 % CI	OR	95 % CI
Demographic factors				
Age	1·040	1·015, 1·066	1·033	1·004, 1·063
Sex, male	0·922	0·528, 1·609	–	–
BMI	1	0·916, 1·091	–	–
Smoker	1·484	0·811, 2·716	–	–
Marital status	1·452	0·238, 8·877	–	–
Past medical history				
Hypertension	1·355	0·732, 2·507	–	–
Diabetes	1·232	0·398, 3·812	–	–
Cerebral infarction	3·725	1·176, 11·806	2·726	0·759, 9·785
Preoperative factors				
NYHA functional class III/IV	2·660	1·349, 5·243	2·410	1·079, 5·383
Intraoperative factors				
Macrovascular surgery	3·565	1·450, 8·764	5·434	1·704, 17·333
Operating time	1·007	1·003, 1·011	1·001	0·993, 1·009
CPB time	1·008	1·003, 1·013	1·001	0·991, 1·011
Aortic cross-clamp time	1·003	0·997, 1·009	–	–
Postoperative factors				
SOFA score	1·445	1·234, 1·693	1·243	1·010, 1·530
FI	2·233	1·207, 4·132	2·138	1·058, 4·320
VIS_max_	1·034	1·008, 1·062	1·012	0·980, 1·045
Lactic acid_max_	1·109	1·026, 1·198	1·002	0·910, 1·104
MAP	1·000	0·984, 1·016	–	–
Anticoagulant therapy	0·605	0·308, 1·190	–	–

NYHA, New York Heart Association; SOFA, sequential organ failure assessment; FI, feeding intolerance; VIS, vasoactive-inotropic score; MAP, mean arterial pressure.

## Discussion

The present study is the first prospective, observational study to evaluate the correlation between FI and poor prognosis in patients undergoing CPB surgery. The results showed that: (1) the incidence of FI after CPB was 64·14 %; (2) FI was significantly associated with prolonged mechanical ventilation (> 48 h), acute kidney injury and neurological events in patients after CPB; and (3) FI, SOFA score, age, NYHA functional class III/IV and macrovascular surgery were independent predictors of poor prognosis after CPB. Therefore, for patients with EN after CPB, assessing FI can help identify patients at risk of poor prognosis.

The systematic review by Blaser *et al.* showed that the incidence of FI was 30·5–65·7 % in critically ill patients and was higher (50·0–88·9 %) in mechanically ventilated patients^(13)^. In this present study, the incidence of FI within 7 d of EN in CPB patients was 64·14 %; the incidence of constipation was high (44·7 %; 106 cases) and the incidence of abdominal distension was low (11·4 %; twenty-seven cases). During CPB, the redistribution of blood flow to critical body organs, such as the brain, results in an earlier reduction in blood flow to abdominal organs. Moreover, other factors, such as haemodilution, cause the gastrointestinal tract to be in a state of low perfusion and hypoxia, which leads to functional gastrointestinal injury^(14)^. Furthermore, in this present study, all the patients underwent mechanical ventilation after CPB. A study^(15)^ has shown that mechanical ventilation can cause bile reflux and gas to enter the stomach, with increased intra-abdominal pressure leading to gastrointestinal dysfunction. Therefore, FI, as the most common clinical manifestation of functional gastrointestinal injury, is easy to occur during the implementation of EN after CPB.

Another significant finding in the present study was the association of FI with poor prognosis in CPB patients. A case–control study^(8)^ on severe neurological diseases found that FI within 3 d of ICU admission was independently associated with poor prognosis in patients with severe neurological diseases. Gungabissoon *et al.* ^(2)^ reported that EN FI in critically ill patients was associated with failed nutritional goals, prolonged duration of mechanical ventilation, increased number of hospitalisation days and 60-d mortality. This present study found that FI was significantly related to a poor prognosis of patients after CPB (OR 2·138; 95 % CI 1·058, 4·320). According to recent research, > 45 % of nurses immediately stopped EN when FI was present^(16)^, resulting in reduced energy and protein intake of 1780·23 kcal (7448·48 kJ) and 100·58 g, respectively^(17)^. Thus, it can be seen that the frequency of EN in patients with FI decreases, or even interrupts feeding, thus leading to nutritional deficiency, which impacts patient prognosis. Poor nutritional status can significantly affect intestinal epithelial cell renewal (proliferation, migration, differentiation and apoptosis) and intestinal barrier function^(18)^, which may lead to impaired digestion and absorption of nutrients, as well as atrophy of lymphoid tissue, decline of immune system function, and an aggravation of intestinal bacterial translocation^(19,20)^. Because of impaired intestinal barrier function and bacterial translocation, the intestinal epithelium and lung epithelium are exposed to high concentrations of foreign antigens, forming the centre for the initiation and maintenance of an inflammatory response, which may lead to or aggravate multiple organ damage or failure^(21)^.

SOFA score is significantly associated with a poor prognosis in patients undergoing CPB. The SOFA score is a scoring system used to evaluate multiple organ dysfunction or failure in ICU patients and is based on the PaO_2_/FiO_2_ ratio, platelet count, bilirubin level, cardiovascular hypotension, nervous system status and renal function. A prospective study of 857 cardiac ICU patients^(22)^ found that the SOFA score was an independent predictor of mortality and hospital stay in patients undergoing cardiac surgery. Moreover, in the early assessment after adult cardiac surgery, the SOFA score was related to morbidity and mortality; the SOFA score on the first day after surgery can predict the 30-d mortality rate^(22)^. In the present study, the multifactor analysis showed that the SOFA score was an independent risk factor for a poor postoperative prognosis of CPB patients (OR 1·243; 95 % CI 1·010, 1·530), which was consistent with the results of previous studies. The incidence of prolonged mechanical ventilation in our study population (> 48 h) was 15·2 %, acute kidney injury 22·4 %, neurological events 5·9 % and death 3·0 %. Compared with data from the Adult Cardiac Surgery Database of the National Association of Thoracic and Cardiac Surgeons in the USA^(23)^, the mortality and prolonged mechanical ventilation (> 48 h) rate in the present study is comparable, but the incidence of acute neurological damage is higher, which may be related to the study population that included patients who had macrovascular surgery. Therefore, in clinical work, patients with high SOFA scores should be closely observed for the function of each organ, and corresponding measures should be taken to reduce the incidence of poor prognosis.

Age and NYHA functional class III/IV were significantly associated with poor prognosis in patients undergoing CPB. Thorsteinsson *et al*. studied 38 830 patients who underwent coronary artery bypass surgery alone and found that with the increase in operation age, the burden of co-morbidities increases, and age is the main predictor of 30-d mortality after surgery^(24)^. An international study found that the mortality rate of type A aortic dissection after surgery increased significantly with age, and age over 70 years was an independent predictor of postoperative mortality^(25)^. At the same time, age is also an influential factor of FI. A previous study has shown that age is also an influential factor for FI in EN patients^(26)^. Our study also found that the age in the FI group was significantly higher than that in non-FI group (54·26 (sd 11·907) *v*. 52·98 (sd 12·863) years). In addition, a study of 638 patients over 80 years of age after aortic valve replacement found that NYHA functional class III/IV was an independent predictor of hospital mortality^(27)^. In the present study, the multivariate analysis showed that age (OR 1·033; 95 % CI 1·004, 1·063) and NYHA functional class III/IV (OR 2·410; 95 % CI 1·079, 5·383) were independent risk factors for poor prognosis in patients with CPB, which was consistent with previous studies. Studies have shown that with age, the cardiovascular system presents progressive structural and functional changes, especially with regard to the decrease in the number of myocardial cells and the increase in interstitial collagen fibres, resulting in impaired left ventricular diastolic function and decreased cardiac function^(28)^. At the same time, the burden of co-morbidities increases due to the decline in the functional reserve of various organs in elderly patients^(24)^. In addition, with the increase in age, jejunal villi become sparse and thick, and the mucosa gradually shrinks, resulting in an increase in the incidence of FI after EN^(26)^. Therefore, we should pay close attention to the occurrence of FI in elderly patients with NYHA functional class III/IV in clinical work and take corresponding measures in time to reduce the incidence of adverse prognosis.

The results of this present study should be interpreted considering the following limitations. First, this project was a single-centre prospective study with a small sample size. A multicentre prospective cohort study with a large sample size is recommended to further verify the predictive value of FI on poor prognosis in CPB patients. Second, we only analysed the results of hospitalised patients and did not follow up patients after discharge. Therefore, we were unable to determine the long-term effect of FI on poor prognosis indicators in patients who had CPB.

### Conclusion

In the present study, FI within 7 d of initiating EN was associated with a poor prognosis (death, gastrointestinal haemorrhage, acute kidney injury, liver insufficiency, nervous system events (cerebral infarction, cerebral haemorrhage and epilepsy) and prolonged mechanical ventilation (> 48 h)) in CPB patients. These results highlight the need for clinicians to focus more on early FI after CPB. However, these results need to be verified in larger prospective studies.
